# Supplementary material for: Induced pluripotent stem cells from human hair follicle keratinocytes as a potential source for in vitro hair follicle cloning
Source: PeerJ. 2016 Nov 10;4:e2695. doi: 10.7717/peerj.2695 (PMC5111897; doi:10.7717/peerj.2695)
Supplement: Data S1 [file peerj-04-2695-s001.docx]

| Pluripotency Markers | iPSC  Clone 1 | iPSC  Clone 2 | iPSC  Clone 3 | Parental Cells (HHFK) |
| --- | --- | --- | --- | --- |
| NANOG | 27.1% | 24.5% | 36.4% | 0.5% |
| OCT4 | 55.6% | 59.1% | 55.9% | 1.7% |
| SOX2 | 60.3% | 64.9% | 63.0% | 1.8% |
| SSEA4 | 98.6% | 95.7% | 98.2% | 0.5% |
| TRA-1-60 | 11.0% | 21.8% | 21.7% | 1.7% |
| TRA-1-81 | 7.2% | 9.6% | 12.8% | 0.4% |

The percentage of pluripotency markers in 3 different iPSC clones and the parental cells, HHFK.

iPSC Clone 1


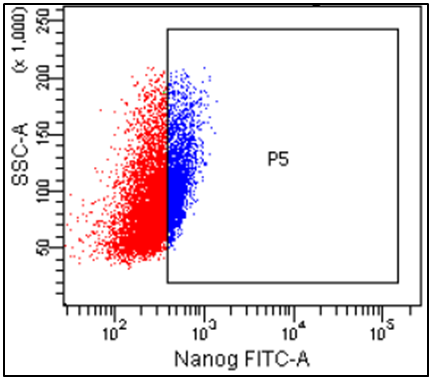

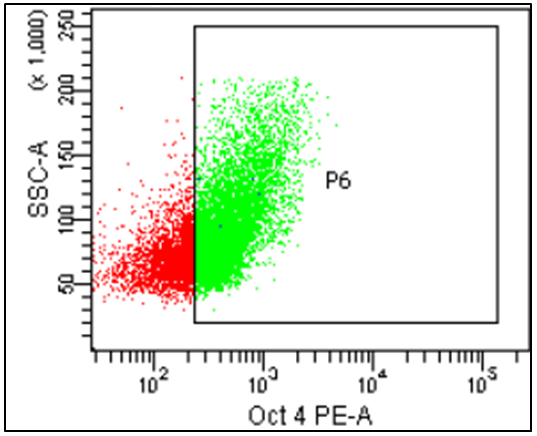

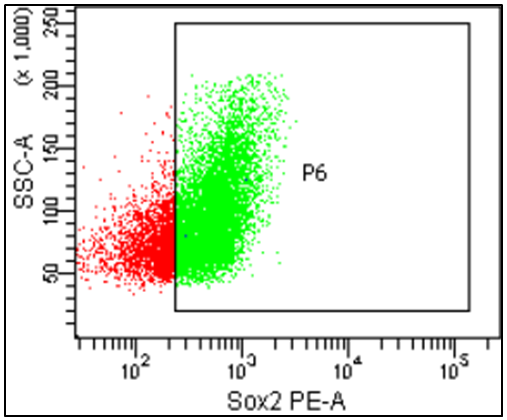


SOX2 (60.3%)

OCT4 (55.6%)

NANOG (27.1%)


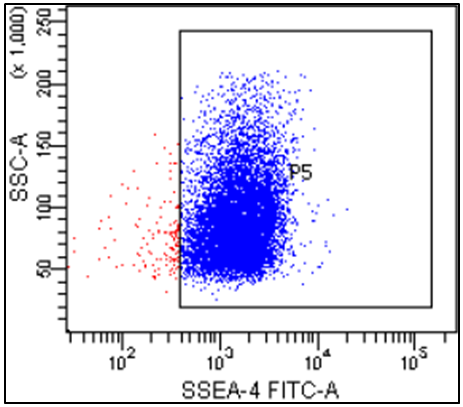

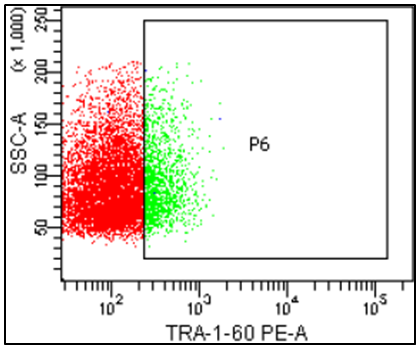

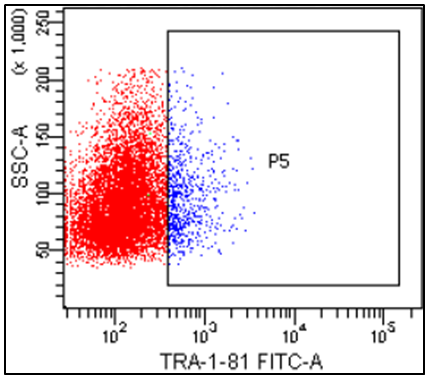


TRA-1-81 (7.2%)

TRA-1-60 (11.0%)

SSEA4 (98.6%)

The dot plot graph obtained from flow cytometry analysis of the pluripotency markers as above for iPSC clone 1.

iPSC Clone 2


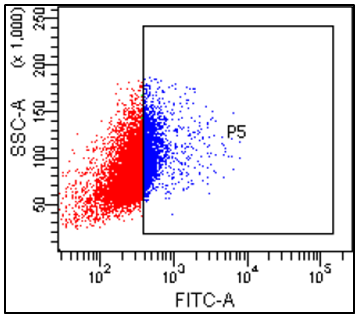

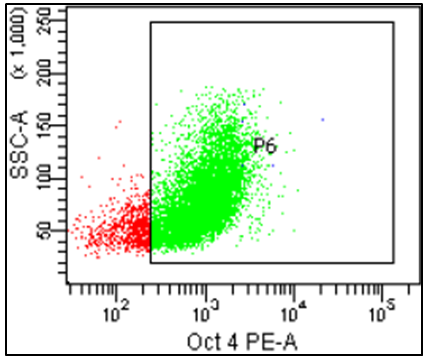

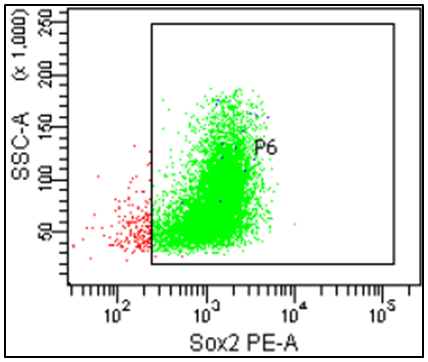


SOX2 (64.9%)

OCT4 (59.1%)

NANOG (24.5%)


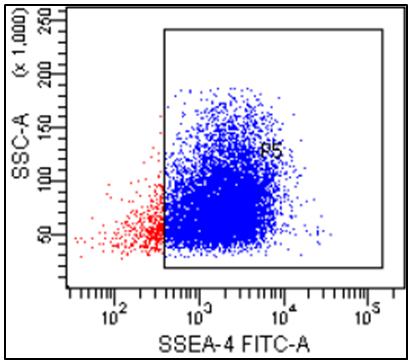

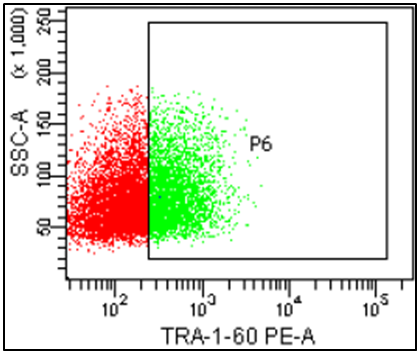

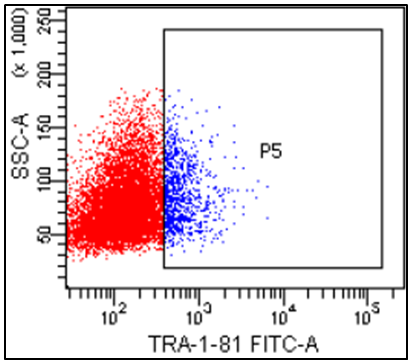


TRA-1-81 (9.6%)

TRA-1-60 (21.8%)

SSEA4 (95.7%)

The dot plot graph obtained from flow cytometry analysis of the pluripotency markers as above for iPSC clone 2.

iPSC Clone 3


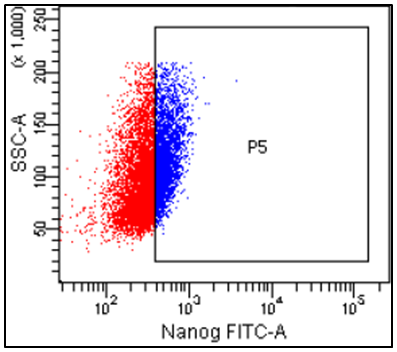

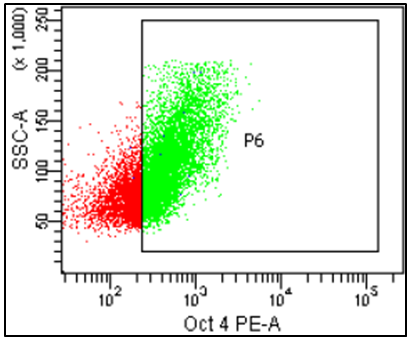

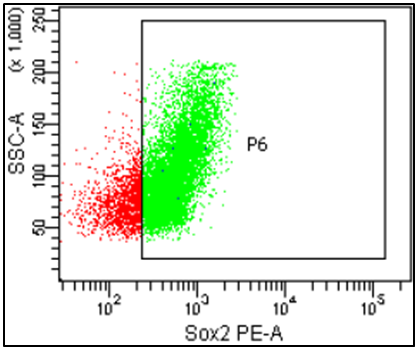


SOX2 (63.0%)

OCT4 (55.9%)

NANOG (36.4%)


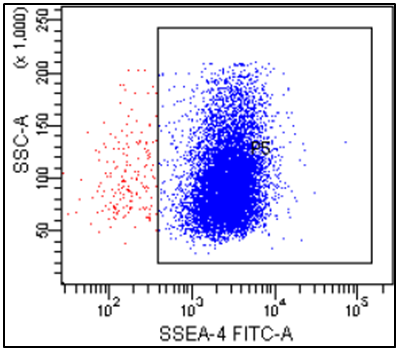

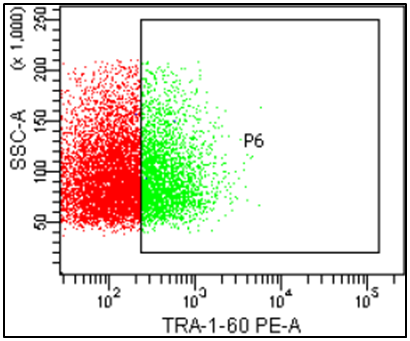

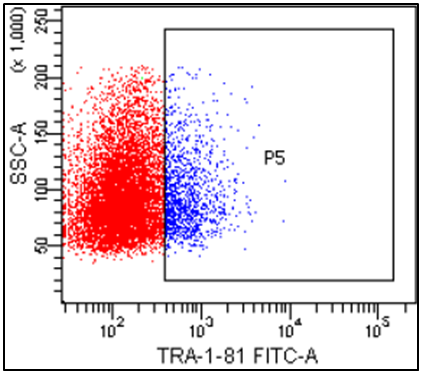


TRA-1-81 (12.8%)

TRA-1-60 (21.7%)

SSEA4 (98.2%)

The dot plot graph obtained from flow cytometry analysis of the pluripotency markers as above for iPSC clone 3.

Parental Cells (HHFK)


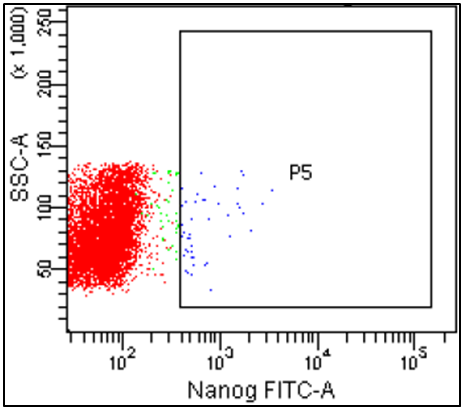

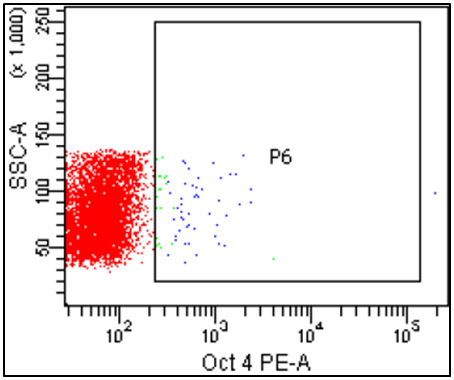

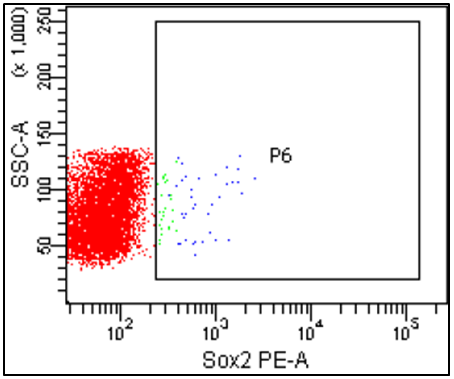


SOX2 (1.8%)

OCT4 (1.7%)

NANOG (0.5%)


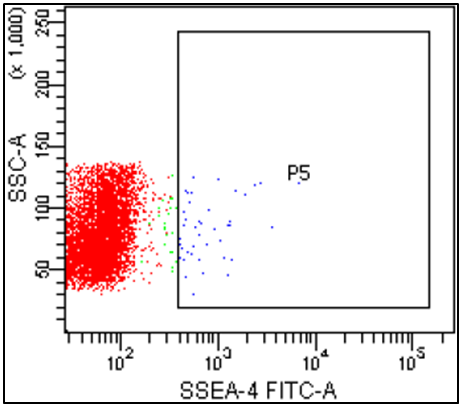

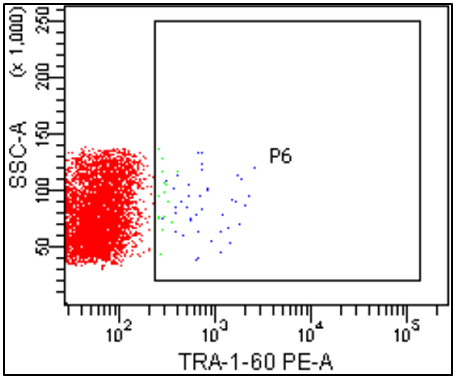

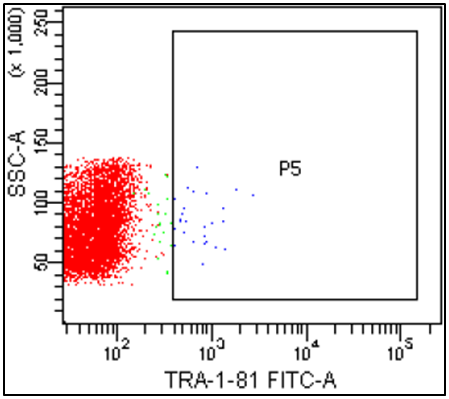


SSEA4 (0.5%)

TRA-1-60 (1.7%)

TRA-1-81 (0.4%)

The dot plot graph obtained from flow cytometry analysis of the pluripotency markers as above for parental cells, HHFK.
